# Pluripotent stem cell model of Shwachman–Diamond syndrome reveals apoptotic predisposition of hemoangiogenic progenitors

**DOI:** 10.1038/s41598-020-71844-8

**Published:** 2020-09-09

**Authors:** Takayuki Hamabata, Katsutsugu Umeda, Kagehiro Kouzuki, Takayuki Tanaka, Tomoo Daifu, Seishiro Nodomi, Satoshi Saida, Itaru Kato, Shiro Baba, Hidefumi Hiramatsu, Mitsujiro Osawa, Akira Niwa, Megumu K. Saito, Yasuhiko Kamikubo, Souichi Adachi, Yoshiko Hashii, Akira Shimada, Hiroyoshi Watanabe, Kenji Osafune, Keisuke Okita, Tatsutoshi Nakahata, Kenichiro Watanabe, Junko Takita, Toshio Heike

**Affiliations:** 1 Department of Pediatrics, Graduate School of Medicine, Kyoto University, 54 Kawahara-cho, Shogoin, Sakyo-ku, Kyoto, 606-8507 Japan; 2 Department of Clinical Application, Center for iPS Cell Research and Application, Kyoto University, Kyoto, 606-8507 Japan; 3 Department of Human Health Sciences, Graduate School of Medicine, Kyoto University, Kyoto, 606-8507 Japan; 4 Department of Cancer Immunotherapy, Osaka University School of Medicine, Suita, 565-0871 Japan; 5 Department of Pediatric Hematology/Oncology, Okayama University, Okayama, 700-8558 Japan; 6 Department of Pediatrics, Graduate School of Biomedical Sciences, Tokushima University, Tokushima, 770-8501 Japan; 7 Department of Cell Growth and Differentiation, Center for iPS Cell Research and Application, Kyoto University, Kyoto, 606-8507 Japan; 8 Department of Life Science Frontiers, Center for iPS Cell Research and Application, Kyoto University, Kyoto, 606-8507 Japan; 9 Drug Discovery Technology Development Office, Center for iPS Cell Research and Application, Kyoto University, Kyoto, 606-8507 Japan; 10 Department of Hematology and Oncology, Shizuoka Children’s Hospital, Shizuoka, 420-8660 Japan

**Keywords:** Differentiation, Induced pluripotent stem cells

## Abstract

Shwachman–Diamond syndrome (SDS), an autosomal recessive disorder characterized by bone marrow failure, exocrine pancreatic insufficiency, and skeletal abnormalities, is caused by mutations in the Shwachman–Bodian–Diamond syndrome (*SBDS*) gene, which plays a role in ribosome biogenesis. Although the causative genes of congenital disorders frequently involve regulation of embryogenesis, the role of the *SBDS* gene in early hematopoiesis remains unclear, primarily due to the lack of a suitable experimental model for this syndrome. In this study, we established induced pluripotent stem cells (iPSCs) from patients with SDS (SDS-iPSCs) and analyzed their in vitro hematopoietic and endothelial differentiation potentials. SDS-iPSCs generated hematopoietic and endothelial cells less efficiently than iPSCs derived from healthy donors, principally due to the apoptotic predisposition of KDR^+^CD34^+^ common hemoangiogenic progenitors. By contrast, forced expression of *SBDS* gene in SDS-iPSCs or treatment with a caspase inhibitor reversed the deficiency in hematopoietic and endothelial development, and decreased apoptosis of their progenitors, mainly via p53-independent mechanisms. Patient-derived iPSCs exhibited the hematological abnormalities associated with SDS even at the earliest hematopoietic stages. These findings will enable us to dissect the pathogenesis of multiple disorders associated with ribosomal dysfunction.

## Introduction

Shwachman–Diamond syndrome (SDS) is a rare autosomal recessive disorder characterized by hematological abnormalities that manifest as cytopenia and progression to myelodysplastic syndrome and acute myeloid leukemia, exocrine pancreatic insufficiency, and skeletal abnormalities^1,2^. Hematopoietic stem cell transplantation is the sole curative treatment to correct the hematological defect of this syndrome, and mortality remains high due to serious post-transplant complications^3^.

Mutations in the Shwachman–Bodian–Diamond (*SBDS*) gene, which is located on chromosome 7q11, are present in approximately 90% of patients with SDS; these mutations commonly arise from gene conversion to the highly similar pseudogene *SBDSP*^4^. *SBDS* mRNA is expressed in a broad range of tissues^4^. Multiple studies have shown that SBDS protein has a primary function in ribosome assembly^5–7^. The additional proposed functions for SBDS, such as mitotic spindle stabilization, chemotaxis, cellular stress responses, and apoptosis, reflect indirect downstream effects of perturbing ribosome assembly^8–13^.

Mutations in genes encoding transcription factors involved in regulating normal development are responsible for a variety of inherited disorders. During embryogenesis, hematopoietic cells (HCs) and endothelial cells (ECs) emerge from common hemoangiogenic progenitors that express vascular endothelial growth factor receptor (VEGFR)-2 (also known as KDR in humans)^14–16^. Indeed, several HC- and/or EC-related transcriptional factors, such as *SCL* and *RUNX1*, are associated with various congenital hematological disorders^17^. Multiple disorders associated with ribosomal dysfunction (so-called ribosomopathies), including SDS and Diamond–Blackfan anemia (DBA), also present with hematological defects^18^; however, the pathogenesis of ribosomopathies has not been fully elucidated. Currently, the field lacks an adequate mouse model of the human disease because the most analogous mutant in mouse fails to faithfully recapitulate all disease-associated phenotype^19–22^: *Sbds*^-/-^ embryos fail to generate HCs and ECs due to early lethality prior to embryonic day (E) 6.5 before both lineages have developed^23^.

Induced pluripotent stem cells (iPSCs) are pluripotent stem cell generated by enforced expression of specific transcription factors^24^. Patient-specific iPSCs, in combination with directed cell differentiation, are a practical source of human embryonic progenitors that can surpass the utility of murine models. Accordingly, these cells have the potential to contribute enormously to patient-oriented research, including disease pathophysiology and drug screening^25^. In this study, we generated iPSCs from three SDS patients and differentiate them into HCs and ECs using our established differentiation system for human embryonic stem cells (ESCs) and iPSCs^26–30^.

## Results

### Generation of iPSCs from SDS patients

**Figure 1:**
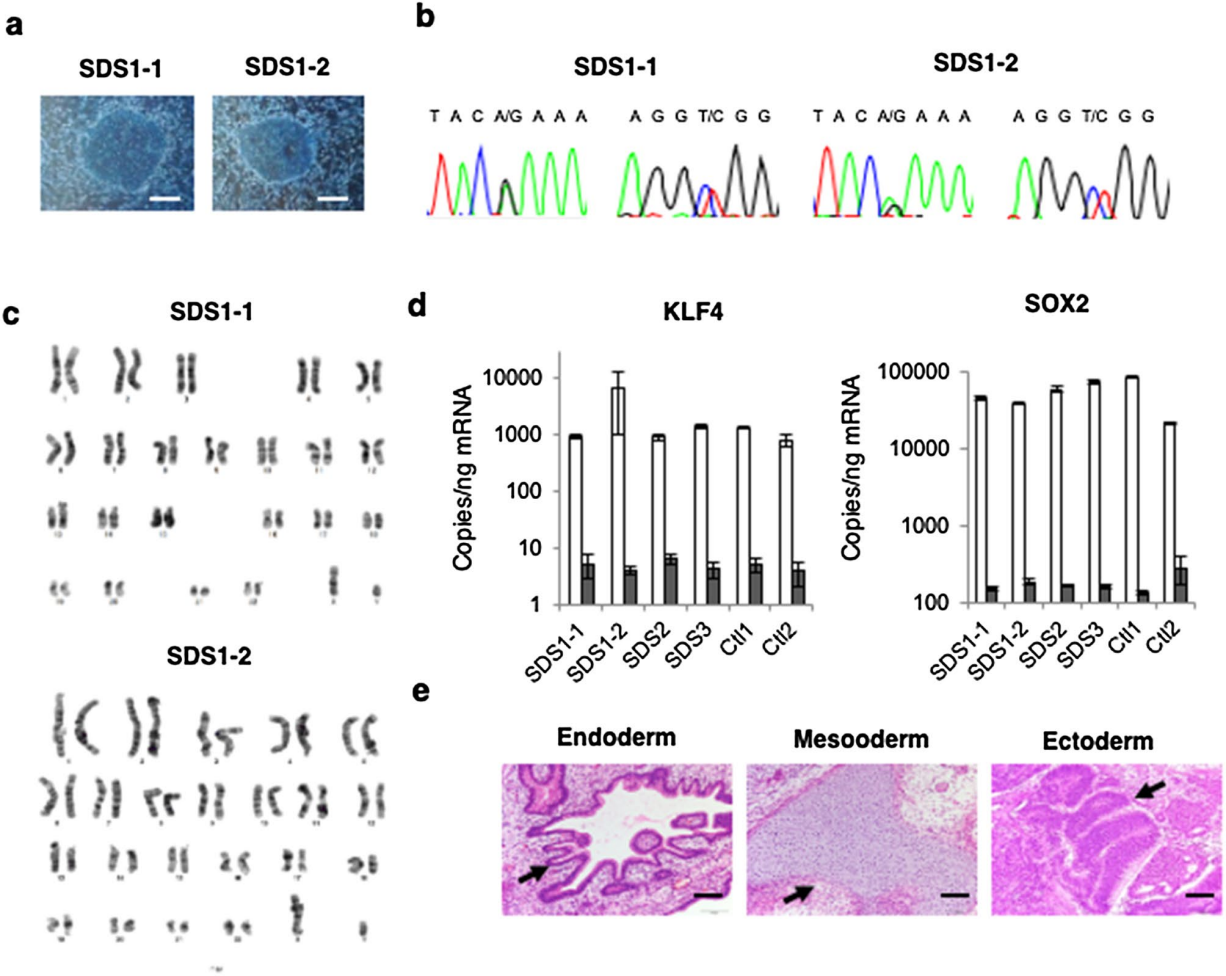
Generation of iPSCs from SDS patients. (**a**) Human ESC-like morphology of SDS-iPSCs. Scale bar: 400 μm. (**b**, **c**) DNA sequencing analysis of *SBDS* RT-PCR product (**b**) and karyotype analysis (**c**) of SDS-iPSCs. (**d**) Expression of *KLF4* and *SOX2* in SDS and control iPSCs. One primer set detects only the transgene (black bars), whereas the other detects both the transgene and endogenous gene (white bars). (**e**) Teratoma formation from SDS-iPSCs in NOD/SCID/γc^null^ mice. Arrows indicate endoderm (respiratory epithelium), mesoderm (cartilage), and ectoderm (pigmented epithelium). Scale bar: 200 μm.

Following transduction of peripheral blood cell derived from SDS patients with an episomal plasmid vector encoding *Oct3/4, Sox2, Klf4, L-Myc, Lin28*, and shRNA against *TP53,* four clones (SDS1-1 and SDS1-2 from patient 1 and SDS2 from patient 2, and SDS3 from patient 3) were randomly selected for propagation and further analyses, as previously reported^29,31^. All patient-derived SDS-iPSCs exhibited a characteristic human ESC-like morphology (Fig. 1a, Supplementary Fig. S1a), and were capable of propagating in serial passage. DNA sequencing analysis verified an identical mutation in the *SBDS* gene in all established SDS-iPSC clones (Fig. 1b, Supplementary Fig. S1b). Chromosomal analysis revealed that all SDS-iPSC clones maintained a normal karyotype (Fig. 1c, Supplementary Fig. S1c). Expression levels of the pluripotency markers *Oct3/4, Sox2, Klf4, L-Myc,* and *Lin28* in all SDS-iPSCs were comparable to those in control iPSCs, although transgene expression was rarely detected (Fig. 1d, Supplementary Fig.S1d). All three primary germ-layer derivatives were detected in cystic teratomas formed after subcutaneous injection of undifferentiated iPSCs into immunocompromised NOD/SCID/γc^null^ mice (Fig. 1e, Supplementary Fig. S1e).

**Figure 2:**
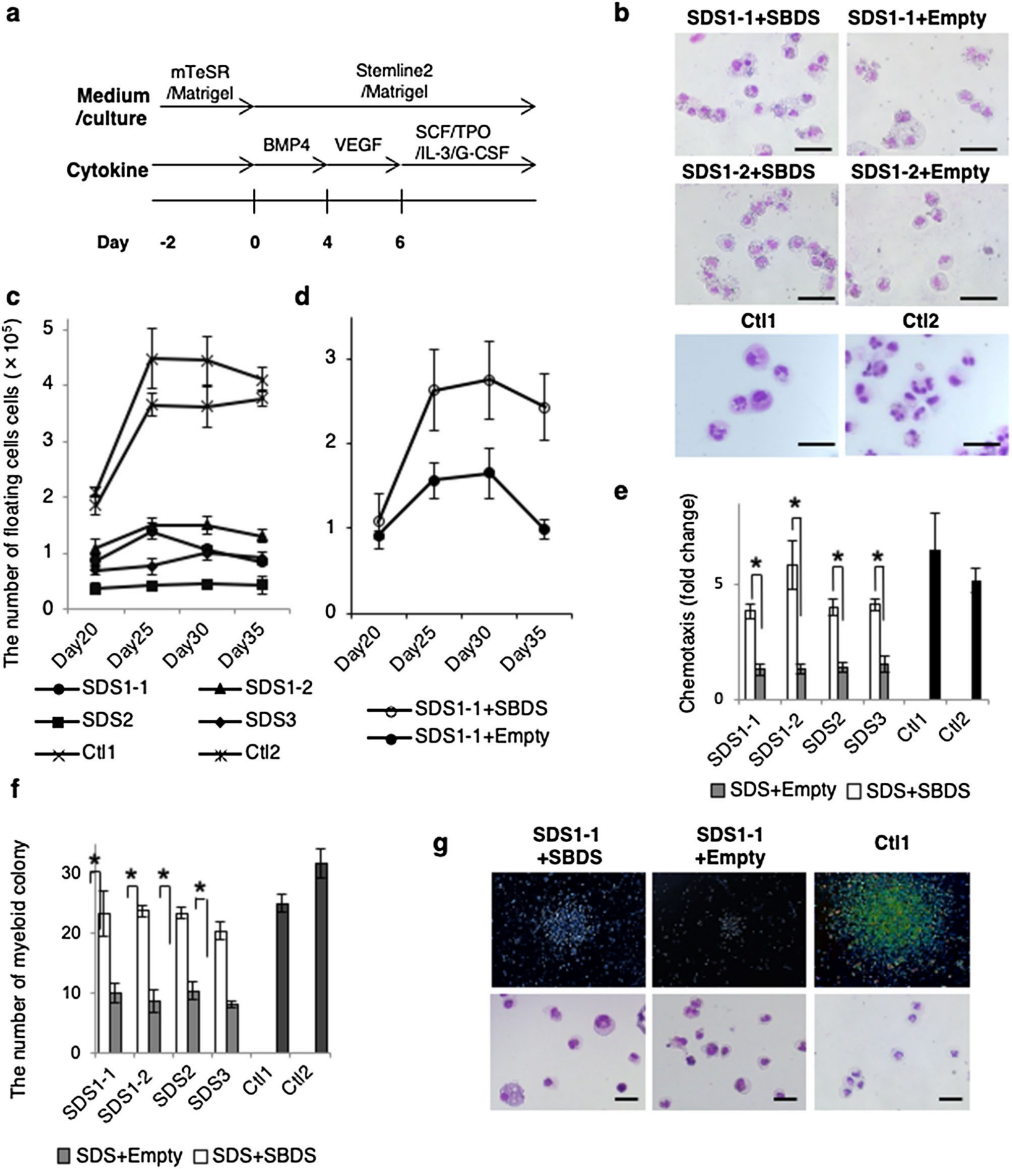
Transduction of SDS-iPSCs. (**a**) Construction of lentiviral vectors containing DsRed alone (PBCl-EF1a-DsRed-PuroR-pA) or SBDS cDNA and DsRed (PBCl-EF1aSBDS-IRES2-DsRed-PuroR-pA). (**b**) Western blotting analysis of SBDS protein in SDS-iPSC clones transduced with SBDS or empty (DsRed alone) vector. TF2B was used as a loading control. “+SBDS” indicates iPSC clones transduced with SBDS vector. (**c**) Representative polysome profiling of SDS-iPSC clones transduced with SBDS or empty vector. (**d**) 80S:40S ratio in SDS-iPSC clones transduced with SBDS or empty vector.

To investigate the pathogenesis of this syndrome, SBDS cDNA and DsRed were transduced into SDS-iPSCs using the PiggyBac transposon system (Fig. 2a). Western blotting revealed a reduction in SBDS protein expression in SDS-iPSCs that was rescued in SBDS-overexpressed iPSCs (Fig. 2b). Polysome profiling demonstrated that ribosomal assembly in SDS-iPSCs was reduced, as evidenced by a decrease in the 80S:40S ratio; this deficiency was reversed by transduction of SBDS cDNA (Fig. 2c,d).

### Impaired granulopoiesis during in vitro differentiation of SDS-iPSCs

**Figure 3:**
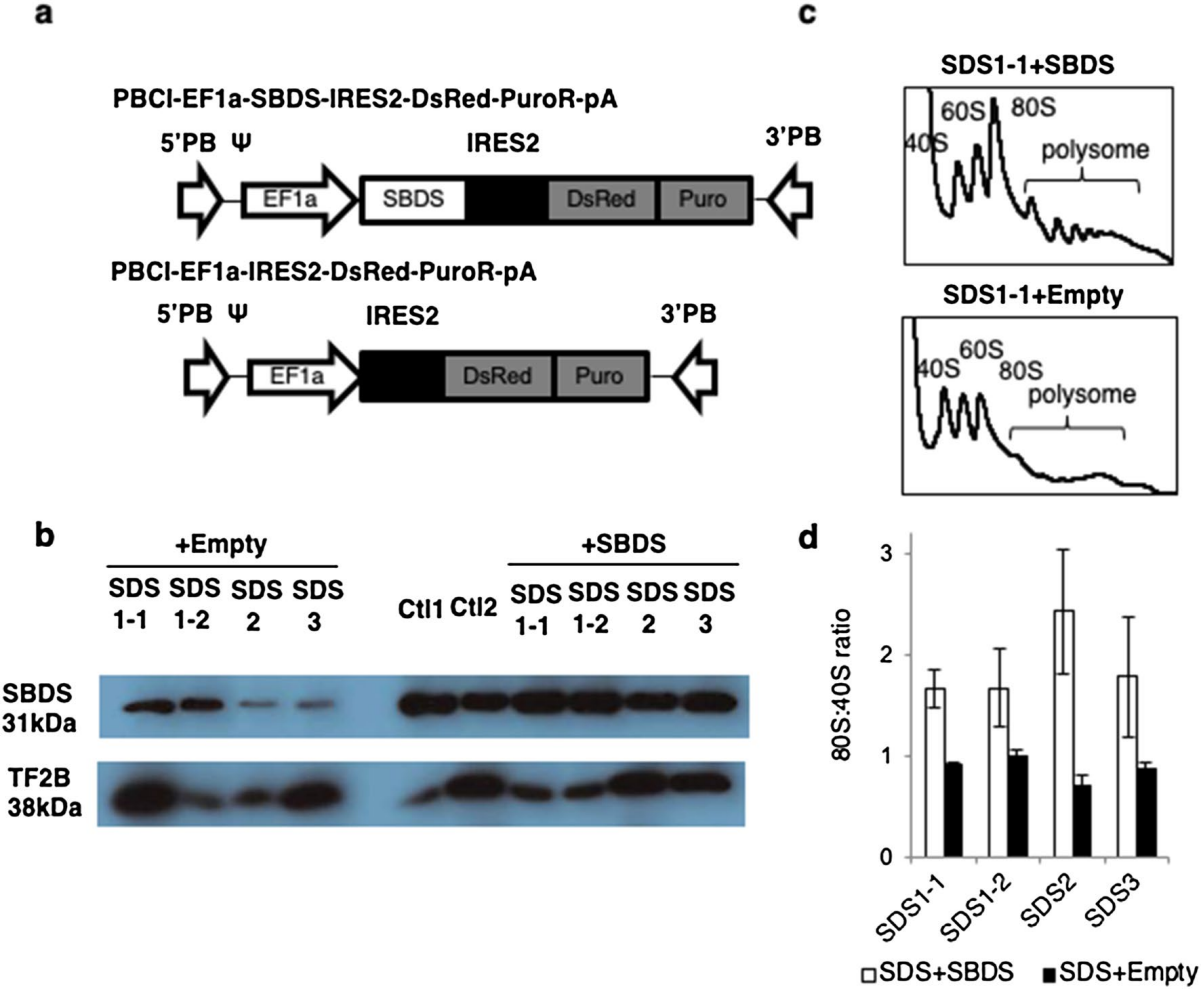
Impaired granulopoiesis during in vitro differentiation from SDS-iPSCs. (**a**) Outline of the defined, step-wise differentiation protocol for generation of mature neutrophils. (**b**) May–Giemsa staining of floating HCs obtained from SDS-iPSC clones transduced with SBDS or empty vector, and from control iPSCs on day 30 of differentiation. Scale bar: 100 μm. (**c**) Sequential analysis of the number of floating HCs generated from control SDS-iPSCs, SBDS-overexpressing SDS-iPSCs, and control iPSCs. (**d**) Sequential analysis of the number of floating HCs generated from SDS-iPSC clones transduced with SBDS or empty vector. (**e**) Chemotactic activity of floating HCs generated from SDS-iPSC clones transduced with SBDS or empty vector in response to fMLP. fMLP-treated samples were presented as the fold change to untreated control sample. (**f**) Number of hematopoietic colonies derived from SDS-iPSC clones transduced with SBDS or empty vector, and from control iPSCs. (**g**) Light micrographs (upper rows) and May–Giemsa staining of hematopoietic colonies (lower rows) generated from SDS-iPSC clones transduced with SBDS or empty vector, and from control iPSCs. Scale bar: 100 μm. Data represent means ± SEM of triplicate wells; representative results from one of three independent experiments are shown (*P < 0.05).

First, using a previously reported in vitro culture system^26,29^, we investigated whether generated SDS-iPSCs recapitulated the hematological phenotype of the syndrome (Fig. 3a). Floating HCs, which mainly consisted of mature neutrophils, first appeared on day 15 of differentiation of SDS and control iPSCs (Fig. 3b,c, Supplementary Fig. S2a). The remaining HCs consisted of immature myeloid cells and a small number of macrophages. Serial analyses revealed that floating HCs generated from SDS-iPSCs were less abundant than those from control iPSCs (Fig. 3c). Positivity for myeloperoxidase and lactoferrin, the constituent proteins of neutrophil-specific granules, was comparable in neutrophils obtained from SDS-iPSCs and control iPSCs (Supplementary Fig. S2b,c). Similarly, the bactericidal activity of neutrophils from SDS-iPSCs and control iPSCs did not significantly differ (Supplementary Fig. S2d). HC production was comparable between SBDS-overexpressing SDS-iPSCs and control iPSCs (Fig. 3c,d, Supplementary Fig. S1e,f), with no attendant morphological changes (Supplementary Fig. S2g,h). As reported previously^9,32^, the chemotactic activity of SDS-iPSC–derived neutrophils was severely impaired, and this deficiency was reversed by overexpression of SBDS (Fig. 3e).

We then examined the hematological defects of SDS-iPSCs at the clonogenic progenitor level. In methylcellulose colony-forming assays, SDS-iPSCs formed significantly fewer HC colonies, a defect that was rescued by SBDS overexpression (Fig. 3f). The decreased size of HC colonies was also reversed by SBDS overexpression, although the size is not somehow comparable to that of control iPSCs (Fig. 3g). Collectively, these data demonstrated that SDS-iPSCs exhibited reduced HC production, accompanied by impaired neutrophil chemotaxis and limited colony-forming potential, all of which are typical hematological abnormalities of SDS patients.

### Reduced HC and EC generation from SDS-iPSC derived hemoangiogenic progenitors

**Figure 4:**
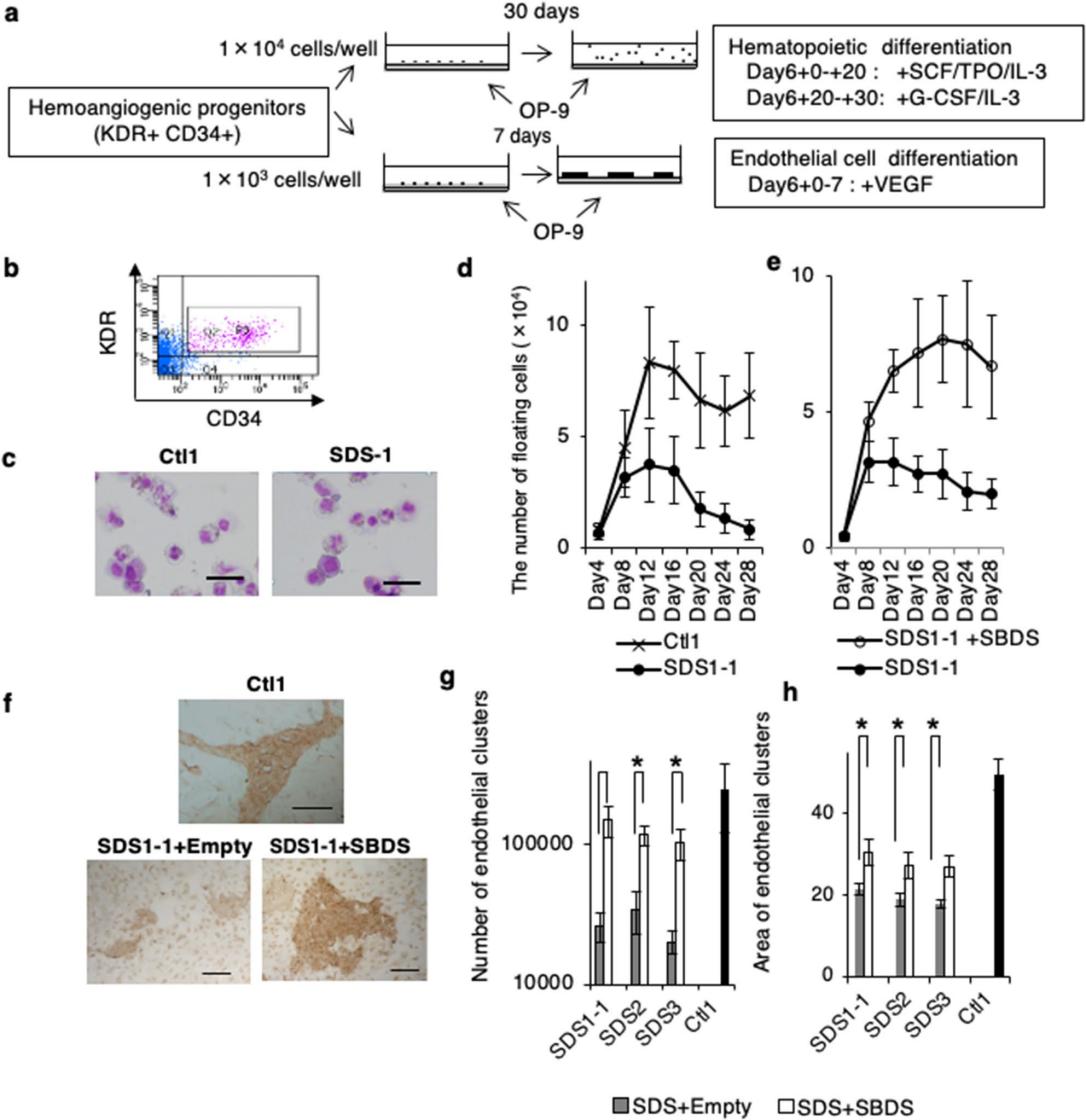
Reduced HC and EC development from SDS-iPSC derived KDR^+^ CD34^+^ hemoangiogenic progenitors. (**a**) Outline of the differentiation protocol for generation of HCs and ECs from iPSC-derived hemoangiogenic progenitors. On day 6 of initial differentiation, sorted KDR^+^CD34^+^ cells were transferred onto fresh confluent OP9 cells, and were then cultured in the indicated culture conditions. (**b**) Isolation of KDR^+^CD34^+^ hemoangiogenic progenitors by FACS. Purities of viable KDR^+^CD34^+^ cells were 7.1% ± 1.3% in at least three independent experiments. (**c**) May–Giemsa staining of floating HCs obtained from SDS and control iPSC–derived KDR^+^CD34^+^ cells on day 26 of differentiation. Scale bar: 100 mm. (**d**, **e**) Sequential analysis of the number of floating HCs generated from SDS and control iPSCs (**d**), and from SDS-iPSC clones transduced with SBDS or empty vector (**e**). (**f**) Immunostaining of CD31 in EC clusters obtained from SDS and control iPSC–derived KDR^+^CD34^+^ cells on day 13 of differentiation. Scale bar: 500 μm. (**g**, **h**) Numbers (**g**) and area (**h**) of EC clusters generated from SDS-iPSC clones transduced with SBDS or empty vector, and from control iPSCs. Data represent means ± SEM of triplicate wells; representative results from one of three independent experiments are shown (*P < 0.05).

To investigate when the initial pathological events occurred during hematopoietic differentiation of SDS-iPSCs, we focused on the development and differentiation potential of KDR^+^CD34^+^ cells (Fig. 4a,b), which represent hemoangiogenic progenitors^26,30^. On day 6 of initial differentiation, a similar proportion of KDR^+^CD34^+^ cells were generated from SDS-iPSCs and control iPSCs (data not shown). After replating onto OP9 cells, however, the KDR^+^CD34^+^ cells generated from SDS-iPSCs produced fewer floating HCs than those generated from control iPSCs, a phenotype reversed by SBDS overexpression (Fig. 4c–e, Supplementary Fig. S3a).

We also investigated the capacity of KDR^+^CD34^+^ cells to differentiate into ECs. KDR^+^CD34^+^ cells generated from SDS-iPSCs produced significantly fewer CD31^+^ EC clusters than those from control iPSCs (Fig. 4f,g). Furthermore, each EC cluster derived from SDS-iPSCs was smaller (Fig. 4h), suggesting reduced EC differentiation or growth potential. The lower EC production and smaller EC cluster size of SDS-iPSCs were rescued by SBDS overexpression (Fig. 4g,h). EC clusters generated from SDS-iPSCs took up 1,1′-dioctadecyl-1,3,3,3′,3′-tetramethylindocarbocyanine–labeled acetylated low-density lipoprotein (Dil-Ac-LDL); co-expressed various EC-related surface markers, including VE-cadherin, CD31, CD34, CD141, CD146, and KDR; and formed tube networks on Matrigel, similarly to those generated from control iPSCs (Supplementary Fig. S3b–d). Thus, KDR^+^CD34^+^ cells generated from SDS-iPSCs had less EC differentiation potential than those generated from control iPSCs, although EC immunophenotype or function did not differ.

### Apoptotic predisposition of SDS-iPSC–derived hemoangiogenic progenitors

**Figure 5:**
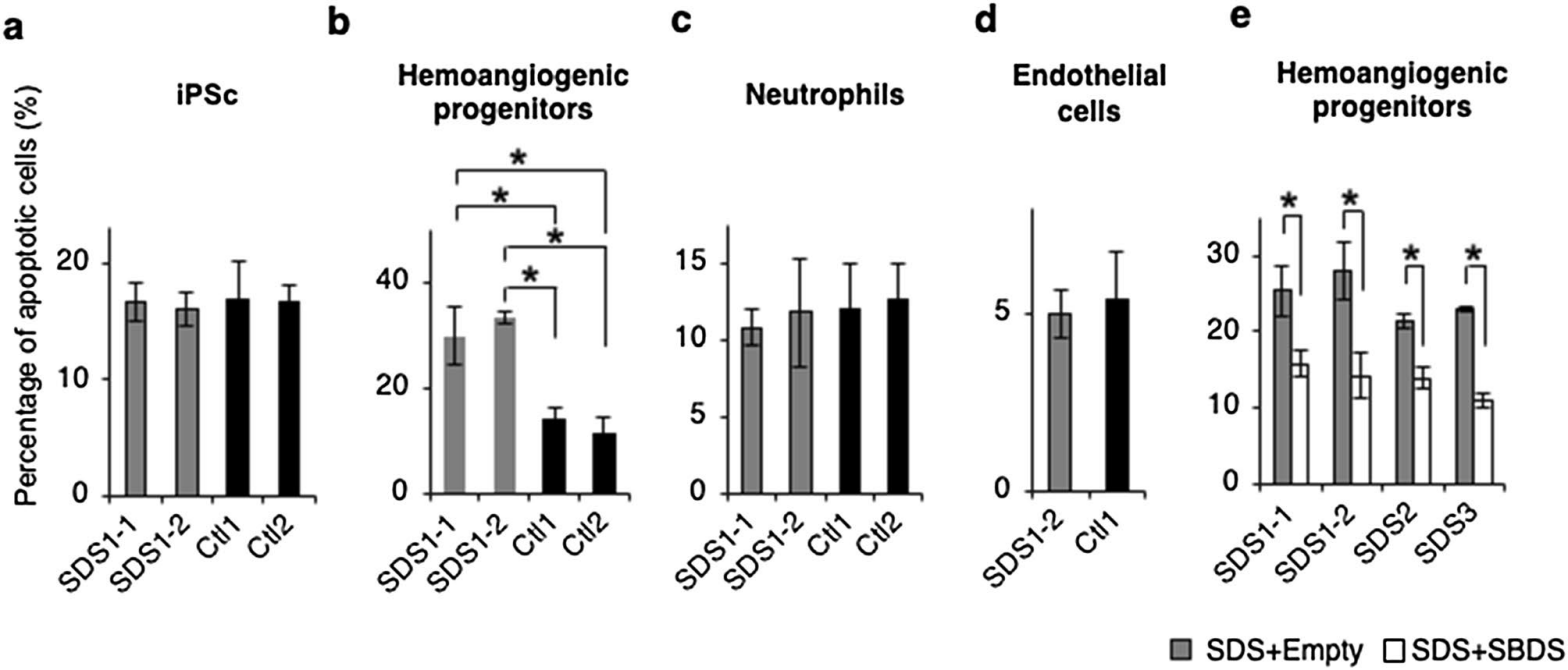
Apoptotic predisposition of SDS-iPSC–derived hemoangiogenic progenitors. (**a**–**d**) Proportion of apoptotic cells in undifferentiated SDS and control iPSCs (**a**) and their derived hemoangiogenic progenitors (**b**), neutrophils (**c**), and EC cells (**d**). (**e**) Proportion of apoptotic cells among hemoangiogenic progenitors generated from SDS-iPSC clones transduced with SBDS or empty vector. Data represent means ± SEM of triplicate wells (*P < 0.05).

Given that elevated apoptosis of hematopoietic stem/progenitor cells has been reported in SDS patients^10,11,33^, we investigated the apoptotic predisposition of hemoangiogenic progenitors or mature cell populations generated during SDS-iPSC differentiation. Detection of caspase 3 and 7 in each population revealed higher proportion of apoptotic cells in SDS-iPSC–derived KDR^+^CD34^+^ cell fraction (Fig. 5b). By contrast, we observed no significant changes in the rate of apoptosis in undifferentiated SDS-iPSCs or the neutrophils and ECs derived from them (Fig. 5a,c,d, Supplementary Fig. S4a–c). Overexpression of SBDS of SDS-iPSC–derived KDR^+^CD34^+^ cells attenuated the increase in apoptosis (Fig. 5e), suggesting that quantitative differences in SBDS expression were directly linked to the apoptotic predisposition of early hematopoietic progenitors. Bromo-2′-deoxyuridine (BrdU) incorporation assays revealed no difference in proliferation of KDR^+^CD34^+^ cells between SDS and control iPSCs (Supplementary Fig. S4d,e). Thus, elevated apoptosis of KDR^+^CD34^+^ hemoangiogenic progenitors is primarily responsible for the reduction in HC and EC development from SDS-iPSCs.

**Figure 6:**
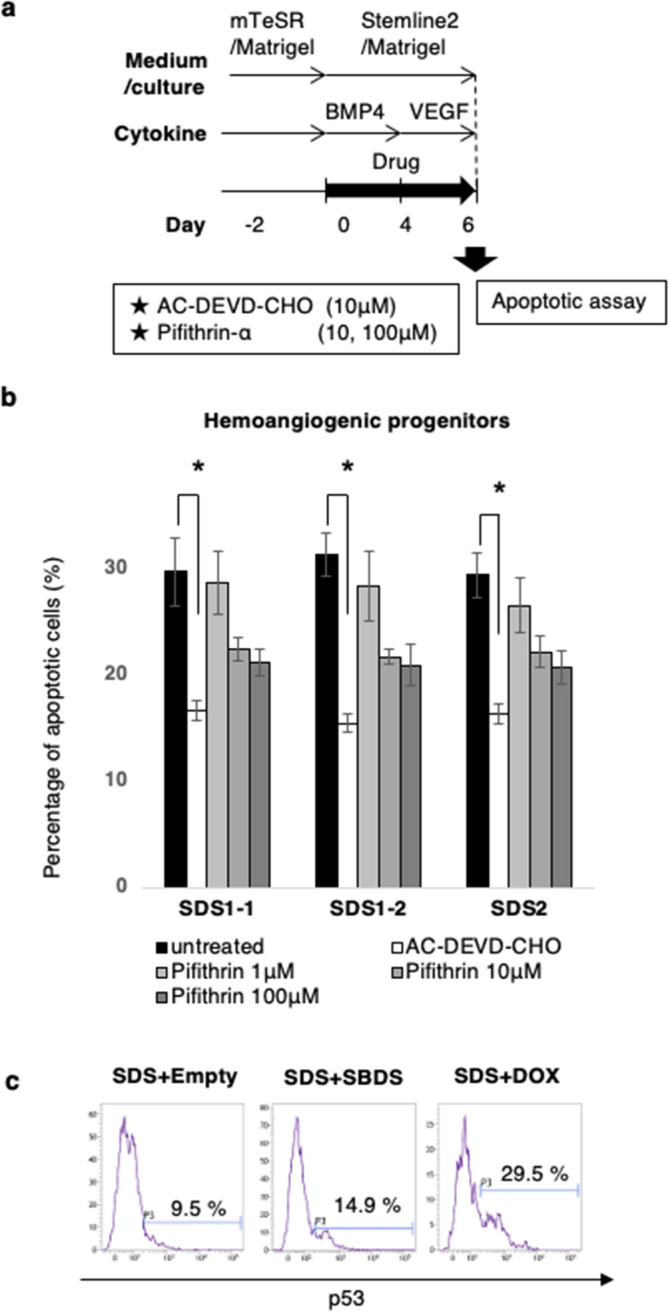
Effect of caspase or p53 inhibitor on the apoptosis of SDS-iPSC–derived hemoangiogenic progenitors. (**a**) Graphical representation of the experimental procedure. (**b**) Proportion of apoptotic cells in untreated (black bars), and AC-DEVD-CHO–treated (white bars) or pifithrin-a–treated SDS-iPSC–derived hemoangiogenic progenitors (gray bars). (**c**) Representative phospho-flow cytometric plot of p53 in SDS-iPSC clones transduced with SBDS or empty vector. Doxorubicin (DOX)-treated SDS-iPSCs was used as a positive control. Data represent means ± SEM of triplicate wells (*P < 0.05).

We then investigated the underlying mechanism of the SDS-associated apoptotic predisposition at the hemoangiogenic progenitor stage (Fig. 6a). Flow cytometric analyses revealed that addition of caspase-3 inhibitor (Ac-DEVD-CHO) significantly decreased the proportion of apoptotic KDR^+^CD34^+^ cells (Fig. 6b), indicating that apoptosis of hemoangiogenic progenitors was caspase-dependent. Activation of p53 has been proposed as a common mechanism in the pathogenesis of various ribosomopathies, including DBA and SDS^21,34^. However, the inhibitory effect of p53 inhibitor (pifithrin-α) was smaller than that of Ac-DEVD-CHO (Fig. 6b). Furthermore, phospho-flow cytometry did not detect higher level of p53 phosphorylation in SDS-iPSC–derived KDR^+^CD34^+^ cells (Fig. 6c), suggesting that p53-independent apoptosis was at least partially involved in apoptotic predisposition of hemoangiogenic progenitors (Fig. 6d).

## Discussion

In this study, our in vitro culture system faithfully recapitulated typical hematological abnormalities in SDS patients, including as impaired granulopoiesis, reduced colony-forming potential, and neutrophil chemotaxis dysfunction, in SDS-iPSC clones from patients harboring different *SBDS* mutations. Furthermore, SBDS overexpression could reverse all disease-related phenotypes, confirming strongly loss of SBDS protein is directly responsible for the hematological defects associated with this syndrome, as reported previously^35^.

It remains unclear when the initial pathological events leading to hematological defects occur in SDS, primarily because the embryonic lethality of *SBDS* knock-out mice occurs prior to HC and EC development^23^, and also due to the ethical restriction for experimental use of human embryonic tissues. This is a first report showing the possibility that the hematopoietic consequences in SDS patients originate from the KDR^+^CD34^+^ early hemoangiogenic progenitor stage. Marked reduction of HC and EC development was principally due to the apoptotic predisposition of KDR^+^CD34^+^ cells. Previous studies showed that SBDS-deficient cells undergo accelerated apoptosis through the Fas-mediated and generally p53-dependent pathway^10,11,33^. By contrast, the current study suggested that apoptosis of KDR^+^CD34^+^ cells was at least partially p53-independent, which has recently advocated as a different pathway in response to impaired ribosome biogenesis^36^.

SDS-iPSC–derived KDR^+^CD34^+^ cells produced significantly fewer EC clusters of smaller sizes, suggesting that EC differentiation or growth was also impaired. By contrast, the impaired EC development of SDS-iPSCs is inconsistent with clinical observations that SDS patients do not have vascular abnormalities^37^. One possible explanation for this discrepancy is that *SBDS* and vasculogenesis-related transcriptional factors are functionally redundant. Alternatively, SDS patients who express detectable levels of SBDS protein expression (see Fig. 2b), as previously reported^38^, might not display vascular abnormalities postnatally. Nonetheless, patient-specific iPSC technology enables us to obtain transient embryonic progenitors, which allows us to analyze the effect of early hematopoiesis in a variety of congenital hematological disorders.

The effects of proteins related to ribosome biogenesis on early hematopoiesis were recently investigated in zebrafish^39,40^ and a human iPSC model^41^. In agreement with the results of this study, Garçon et al. recently reported a significant decrease in the abundance of CD43^+^ HC progenitors during *RPS19*-mutated DBA-iPSC differentiation^41^, largely consistent with the general assumption that loss of ribosome biogenesis principally affects rapidly proliferating progenitors with high translation rates. Notably, SDS-iPSC–derived neutrophils exhibited significantly decreased chemotaxis, whereas no functional differences were observed in ECs generated from SDS-iPSCs and control iPSCs. Thus, although the *SBDS* gene is ubiquitously expressed in all derivative cells, mutations in this gene can lead to cell- and tissue-specific development or maturation defects, as previously described^42^.

In conclusion, our results obtained from SDS patient–derived iPSCs revealed that disease onset might occur as early as early hematopoiesis when hemoangiogenic progenitors first emerge. This culture system will serve as a new tool to facilitate disease modeling, drug screening, and cell therapy for ribosomopathies.

## Material and methods

### Patients

This study was approved by the Ethics Committee of Kyoto University, and informed consent was obtained from a parent and/or legal guardians in accordance with the Declaration of Helsinki. To generate patient-derived iPSCs, peripheral blood cells were obtained from three male SDS patients with the three characteristic clinical signs of the disease (pancytopenia, exocrine pancreatic insufficiency, and short stature). Each patient was a compound heterozygote harboring a splice site mutation of intron 2 (258 + 2 T > C) in conjunction with another previously reported mutation in *SBDS* (97A > G in patient 1, 184A > T in patient 2, and 183-184TA > CT in patient 3)^4,43^. Control iPSCs were generated from parents of patient 1 (Ctl1 from the father and Ctl2 from the mother), who carried single mutant alleles and were asymptomatic.

### Generation and characterization of SDS-iPSCs

Patient-derived iPSCs were generated as previously reported^29,31^. In brief, *Oct3/4, Sox2, Klf4, L-Myc, Lin28,* and shRNA against *TP53* were introduced into peripheral blood cells obtained from three SDS patients or their parents using an episomal plasmid vector. The transduced cells were harvested and re-plated onto SNL feeder cells treated with mitomycin C (Kyowa Hakko Kirin). Colonies similar to human ESCs were selected for further cultivation in primate ESC medium (ReproCELL, Yokohama, Japan) supplemented with 5 ng/ml bFGF (R&D Systems). Three weeks later, individual colonies were isolated and expanded. Human iPSCs were maintained on mitotically inactive SNL feeder cells and subcultured onto new SNL feeder cells every 7 days, as described previously^22^. Culture conditions, characterization of iPSC stemness and pluripotency profile, karyotyping analysis, DNA sequencing analysis, and teratoma formation were performed as previously reported^28,29^.

### Gene transfer in SDS-iPSCs using the PiggyBac transposon system

The CRA236-EF1α-SBDS-IRES2-DsRed-Puro vector was expressed and purified according to a recently published protocol^31^. SDS-iPSCs were incubated with 10 μM Y-27632 (Wako, Saitama, Japan) for 1 h, and then treated with 0.25% trypsin (Thermo Fisher Scientific, Waltham, MA, USA), 1 mg/ml collagenase IV (Thermo Fisher Scientific), and Accumax (Innovative Cell Technologies, San Diego, CA, USA). The resultant single cells were transduced with the SBDS expression vector and transposase expression vector by electroporation, and then seeded onto fresh mitomycin C–treated SNL76/7 feeder cells in human ESC medium containing 10 μM Y-27632 and 1 μg/ml puromycin (Sigma-Aldrich, St. Louis, MO, USA). One week later, small iPSC colonies were manually picked up and seeded onto fresh mitomycin C–treated SNL76/7 feeder cells. Ectopic expression of SBDS protein in isolates of transduced iPSCs was verified by western blotting analysis.

### Initial differentiation of iPSCs using a serum- and feeder-free culture system

Initial differentiation was performed, as previously described^29^. Briefly, iPSC colonies were cultured on culture dishes coated with growth factor–reduced Matrigel (Becton–Dickinson), in Stemline II hematopoietic stem cell expansion medium (Sigma-Aldrich) containing the insulin–transferrin–selenium (ITS) supplement (Thermo Fisher Scientific) and 20 ng/ml BMP4 (R&D Systems). VEGF (40 ng/ml, R&D Systems) was added on day 4, and the cells were cultivated for 2 more days. To induce neutrophil differentiation, VEGF was replaced with a combination of 50 ng/ml SCF (R&D Systems), 50 ng/ml IL-3 (R&D Systems), 5 ng/ml TPO (R&D Systems), and 50 ng/ml G-CSF (Kyowa Hakko Kirin) on day 6. Thereafter, the medium was replaced every 5 days.

### FACS analysis and cell sorting

Staining procedures, FACS analysis, and cell sorting were performed as reported previously^27,29^. Briefly, cultured cells were harvested with Accumax (Innovative Cell Technologies) and incubated with the indicated primary antibodies for 30 min. Non-viable cells were excluded from analysis by DAPI co-staining. FACS analysis was performed on a Verse flow cytometer with the FACSuite software (Becton–Dickinson). Cell sorting with PE-conjugated CD34 and APC-conjugated VEGFR-2 mAbs was performed on an ARIA II flow cytometer (Becton–Dickinson), as reported previously^27,29^.

### In vitro HC and EC differentiation of hemoangiogenic progenitors

KDR^+^CD34^+^ cells were sorted by FACS on day 6 of initial differentiation in a serum-and feeder-free culture system, as previously described^26,29^. In some experiments, 10 μM Ac-DEVD-CHO (Peptide Institute, Osaka) or 50 μM pifithrin-α (Wako) was added on day 0.

To induce HC and EC differentiation, the sorted KDR^+^CD34^+^ cells were then transferred onto fresh confluent OP9 cells^26,30^. For HC development, the sorted cells were cultured in α-MEM (Thermo Fisher Scientific) supplemented with 10% fetal calf serum (FCS) (Thermo Fisher Scientific), 50 μM 2-mercaptoethanol (ME) (Wako), 10 ng/ml thrombopoietin (TPO) (R&D Systems, Minneapolis MN, USA), 20 ng/ml interleukin-3 (R&D Systems), and 100 ng/ml stem cell factor (SCF) (R&D Systems) until day 20 of differentiation. Subsequently, 10 ng/ml granulocyte colony-stimulating factor (G-CSF, Kyowa Hakko Kirin, Tokyo, Japan) was added instead of SCF and TPO. Methylcellulose colony-forming assays were performed as previously reported^29^. To analyze EC development, the sorted cells were cultured in α-MEM supplemented with 10% FCS, 50 μM 2-ME, and 20 ng/ml VEGF (R&D Systems).

### Characterization of HCs

Serially collected cells were selected using the Dead Cell Removal Kit (Miltenyi Biotec, Bergisch Gladbach, Germany), centrifuged onto glass slides using a Shandon Cytospin® 4 cytocentrifuge (Thermo Fisher Scientific), fixed immediately with PBS containing 4% paraformaldehyde, subjected to May–Giemsa and myeloperoxidase staining and immunostaining for lactoferrin, and analyzed by microscopy, as previously reported^27,29^.

Chemotaxis was determined using a modified Boyden chamber method, as previously reported^20^. Briefly, 500 μl of the reaction medium (Hank’s Balanced Salt Solution containing 2.5% FCS) with or without 10 nM formyl-Met-Leu-Phe (fMLP; Sigma-Aldrich) was placed into each well of a 24-well plate, and the cell culture insert (3.0-mm pores; Becton Dickinson) was gently placed into each well to divide the well into upper and lower sections. Floating cells were suspended to the upper well at a concentration of 3.5 × 10^4^ cells per well, allowing the cells to migrate from the upper to the lower side of the membrane for 4 h at 37 °C. After incubation, cells in the lower chamber were collected and counted by flow cytometry. Dihydrorhodamine assay was performed as previously reported^27^.

### Characterization of ECs

Six days after sorting, cells were stained with anti–VE-cadherin and HRP-conjugated anti-mouse IgG, and EC clusters were scored by microscopy. Data analysis was performed using the ImageJ software (https://imagej.nih.gov/ij/) to calculate the size of each EC cluster. For the Dil-Ac-LDL (Thermo Fisher Scientific) incorporation assay, adherent cells were incubated with 10 μg/ml Dil-Ac-LDL, as previously reported^44^.

The tube formation assay was performed as previously reported^45^. In brief, cells (1.0 × 10^5^) were seeded on 48-well plates precoated with growth factor–reduced Matrigel, incubated for 24 h at 37℃, and analyzed with the ImageJ software.

### RNA isolation and quantitative reverse transcription polymerase chain reaction (RT-PCR) analysis

RNA was isolated using the RNeasy mini kit (Qiagen, Valencia, CA, USA) and subjected to RT with the Omniscript-RT kit (Qiagen). Quantitative PCR was performed on a 7900HT Fast Real-Time PCR system (Applied Biosystems, Carlsbad, CA, USA) with SYBR *Premix Ex Taq* II (Takara, Shiga, Japan). Primer sequences are provided in Supplementary Table 1.

### Western blot analysis

Cells were lysed, and nuclear fractions of soluble protein were extracted using the NE-PER Nuclear and Cytoplasmic Extraction Reagents Kit (Thermo Fisher Scientific), following the manufacturer’s instructions. Nuclear protein was boiled for 5 min, separated by SDS-PAGE, and transferred to a polyvinylidene fluoride membrane. After blocking with 5% skim milk in TBS-T for 1 h at room temperature, the membrane was incubated overnight at 4℃ with primary antibody diluted in the same solution, washed extensively, and incubated for 1 h at room temperature with secondary antibody diluted in the same solution, followed by chemiluminescence detection.

### Polysome profiling

Approximately 80–90% confluent iPSCs were treated with 100 μg/ml cycloheximide (Sigma-Aldrich) for 10 min, harvested, and lysed in 500 μl hypotonic buffer [1.5 mM KCl, 2.5 mM MgCl_2_, 5.0 mM Tris–Cl (pH 7.5), 100 μg/ml cycloheximide, 1 mM dithiothreitol, 100 units RNase inhibitor, 0.5% Triton X-100, 0.5% sodium deoxycholate, and 1 × protease inhibitor cocktail]. Lysates were centrifuged at 15,000 rpm for 5 min at 4 °C, and the supernatant was loaded onto linear 5–50% sucrose gradients [20 mM HEPES (pH 7.6), 5 mM MgCl_2_, 100 mM KCl, 10 μg/ml cycloheximide, 10 U/ml RNase inhibitor, and 1 × protease inhibitor cocktail] using a GradientStation (BioComp Frederiction, NB, Canada). Gradients were centrifuged at 35,000 rpm for 2 h at 4 °C in a SW40Ti rotor (Beckman Coulter, Indianapolis, IN, USA), and fractionated on an ISCO fractionator.

### Apoptosis assay

To investigate apoptosis, caspases 3 and 7 were detected with the FAM-FLICA in vitro Caspase Detection Kit (ImmunoChemistry TECHNOLOGIES, Bloomington, MN, USA).

### Statistical analyses

Differences in mean values between groups were analyzed by Student’s t-test and a Tukey–Kramer multiple comparison test. Statistical significance was defined as* P* < 0.05.

## Supplementary information

Supplementary Information.
