# Author Correction: Pluripotent stem cell model of Shwachman–Diamond syndrome reveals apoptotic predisposition of hemoangiogenic progenitors

**DOI:** 10.1038/s41598-021-81066-1

**Published:** 2021-01-18

**Authors:** Takayuki Hamabata, Katsutsugu Umeda, Kagehiro Kouzuki, Takayuki Tanaka, Tomoo Daifu, Seishiro Nodomi, Satoshi Saida, Itaru Kato, Shiro Baba, Hidefumi Hiramatsu, Mitsujiro Osawa, Akira Niwa, Megumu K. Saito, Yasuhiko Kamikubo, Souichi Adachi, Yoshiko Hashii, Akira Shimada, Hiroyoshi Watanabe, Kenji Osafune, Keisuke Okita, Tatsutoshi Nakahata, Kenichiro Watanabe, Junko Takita, Toshio Heike

**Affiliations:** 1 Department of Pediatrics, Graduate School of Medicine, Kyoto University, 54 Kawahara-cho, Sakyo-ku, Shogoin, 606-8507 Japan; 2 Department of Clinical Application, Center for iPS Cell Research and Application, Kyoto University, Kyoto, 606-8507 Japan; 3 Department of Human Health Sciences, Graduate School of Medicine, Kyoto University, Kyoto, 606-8507 Japan; 4 Department of Cancer Immunotherapy, Osaka University School of Medicine, Suita, 565-0871 Japan; 5 Department of Pediatric Hematology/Oncology, Okayama University, Okayama, 700-8558 Japan; 6 Department of Pediatrics, Graduate School of Biomedical Sciences, Tokushima University, Tokushima, 770-8501 Japan; 7 Department of Cell Growth and Differentiation, Center for iPS Cell Research and Application, Kyoto University, Kyoto, 606-8507 Japan; 8 Department of Life Science Frontiers, Center for iPS Cell Research and Application, Kyoto University, Kyoto, 606-8507 Japan; 9 Drug Discovery Technology Development Office, Center for iPS Cell Research and Application, Kyoto University, Kyoto, 606-8507 Japan; 10 Department of Hematology and Oncology, Shizuoka Children’s Hospital, Shizuoka, 420-8660 Japan

Correction to: *Scientific Reports* 10.1038/s41598-020-71844-8, published online 09 September 2020

This Article contains an error in the Results section, under subheading ‘Apoptotic predisposition of SDS-iPSC–derived hemoangiogenic progenitors.’

“We then investigated the underlying mechanism of the SDS-associated apoptotic predisposition at the hemoangiogenic progenitor stage (Fig. 6a).”

should read:

“We then investigated the underlying mechanism of the SDS-associated apoptotic predisposition at the hemoangiogenic progenitor stage (Fig. 6a). We confirmed that plasmids encoding shRNA against *TP53* were not integrated in all SDS-iPSC clones used in Fig. 6.”

In addition, there is an error in the order of the Figures. Figures 2 and 3 are published as Figures 3 and 2 respectively. The correct Figures 2 and 3 appear below as Figures 1 and 2. The Figure legends are correct.

**Figure 1:**
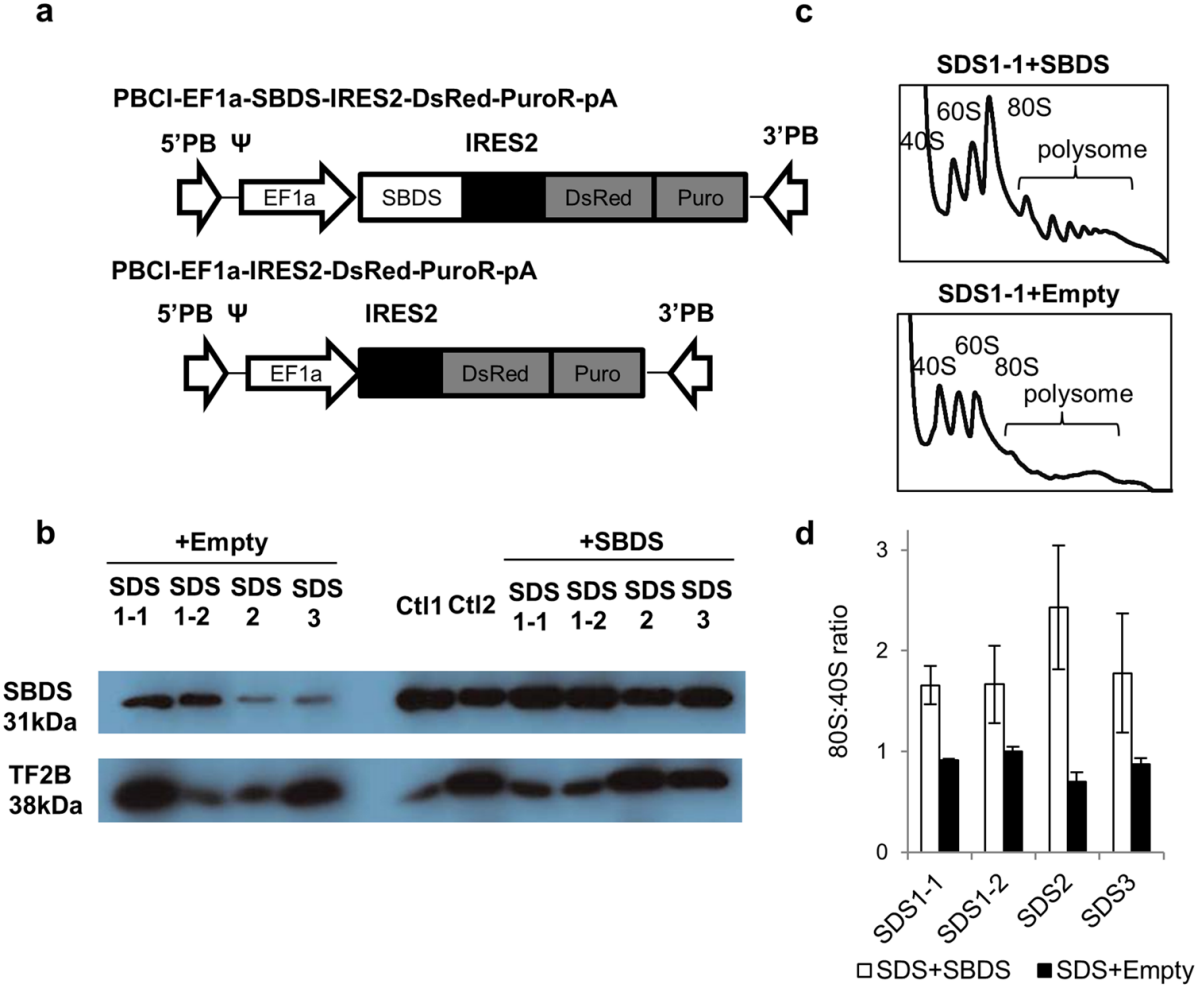
Impaired granulopoiesis during in vitro differentiation from SDS-iPSCs. (**a**) Outline of the defined, step-wise differentiation protocol for generation of mature neutrophils. (**b**) May–Giemsa staining of floating HCs obtained from SDS-iPSC clones transduced with SBDS or empty vector, and from control iPSCs on day 30 of differentiation. Scale bar: 100 μm. (**c**) Sequential analysis of the number of floating HCs generated from control SDS-iPSCs, SBDS-overexpressing SDS-iPSCs, and control iPSCs. (**d**) Sequential analysis of the number of floating HCs generated from SDS-iPSC clones transduced with SBDS or empty vector. (**e**) Chemotactic activity of floating HCs generated from SDS-iPSC clones transduced with SBDS or empty vector in response to fMLP. fMLP-treated samples were presented as the fold change to untreated control sample. (**f**) Number of hematopoietic colonies derived from SDS-iPSC clones transduced with SBDS or empty vector, and from control iPSCs. (**g**) Light micrographs (upper rows) and May–Giemsa staining of hematopoietic colonies (lower rows) generated from SDS-iPSC clones transduced with SBDS or empty vector, and from control iPSCs. Scale bar: 100 μm. Data represent means ± SEM of triplicate wells; representative results from one of three independent experiments are shown (*P < 0.05).

**Figure 2:**
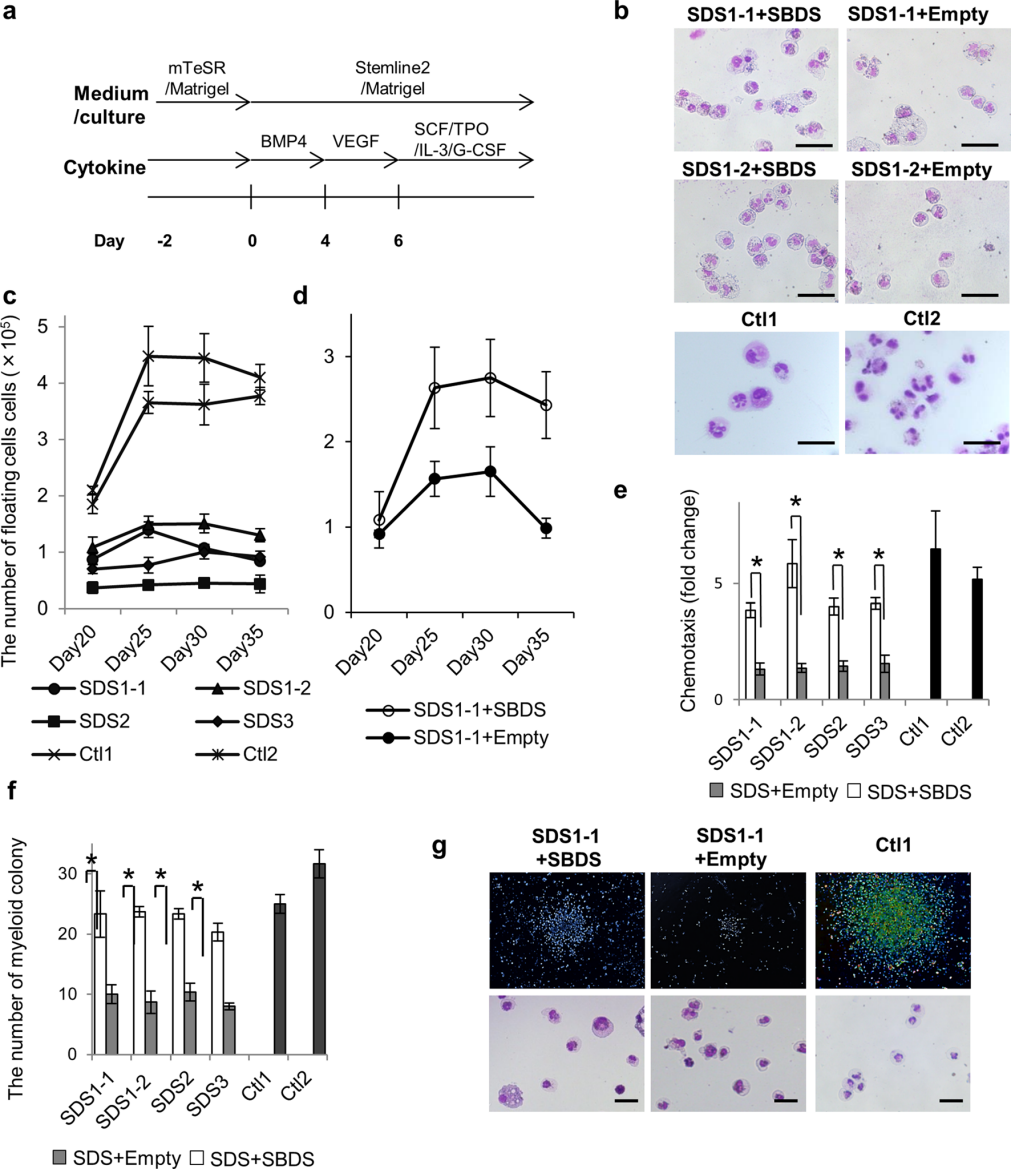
Transduction of SDS-iPSCs. (**a**) Construction of lentiviral vectors containing DsRed alone (PBCl-EF1a-DsRed-PuroR-pA) or SBDS cDNA and DsRed (PBCl-EF1aSBDS-IRES2-DsRed-PuroR-pA). (**b**) Western blotting analysis of SBDS protein in SDS-iPSC clones transduced with SBDS or empty (DsRed alone) vector. TF2B was used as a loading control. “ + SBDS” indicates iPSC clones transduced with SBDS vector. (**c**) Representative polysome profiling of SDS-iPSC clones transduced with SBDS or empty vector. (**d**) 80S:40S ratio in SDS-iPSC clones transduced with SBDS or empty vector.

Finally, in the Supplementary Information file, the labels for the original Western blots for Figure 2b are reversed. The correct version of the blots appears below as Figure 3.

**Figure 3:**
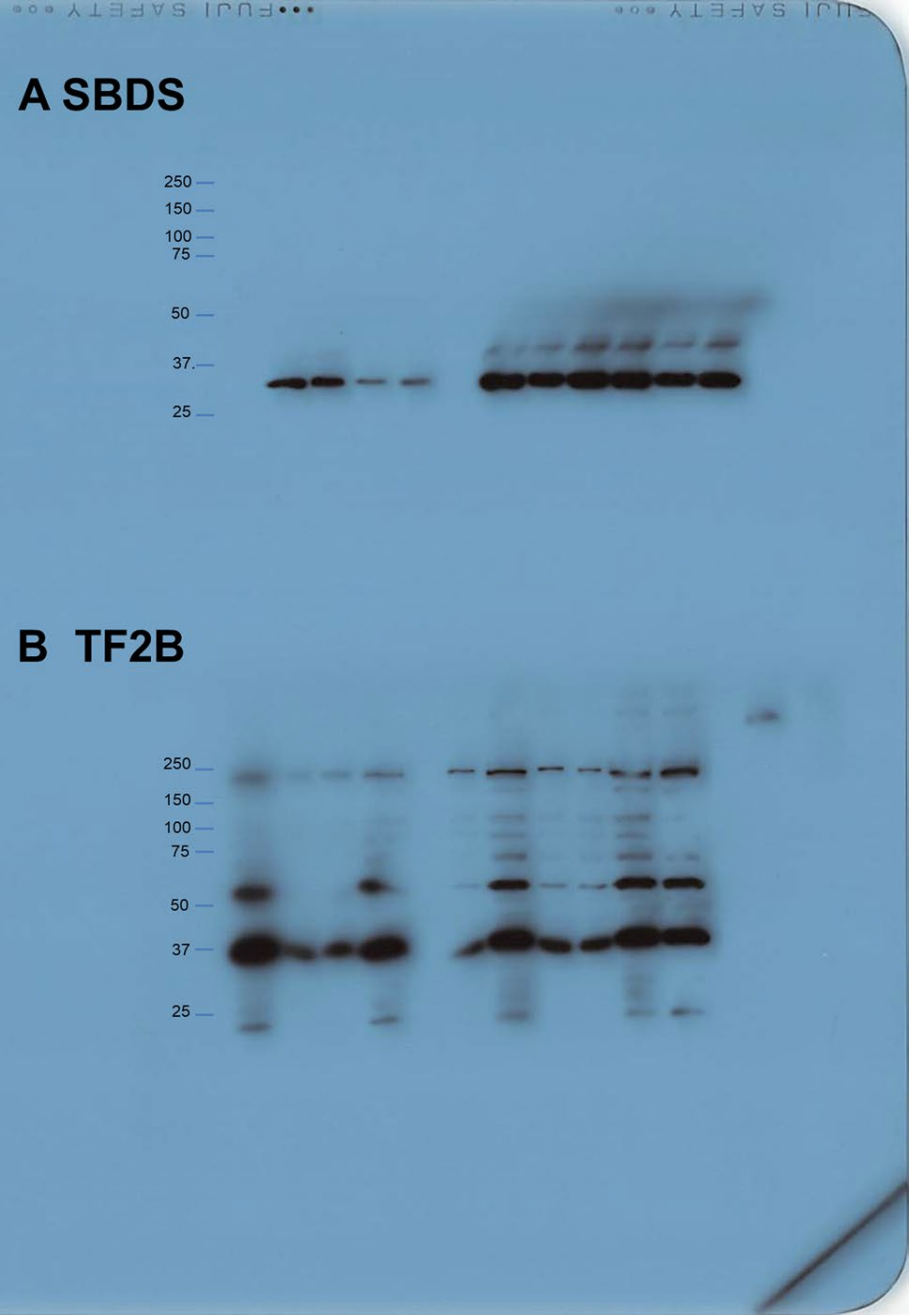
A correct version of the original Western blots for Figure 2b in the Supplementary Information file.

