# Supplementary material for: Pluripotent stem cell model of Shwachman–Diamond syndrome reveals apoptotic predisposition of hemoangiogenic progenitors
Source: Sci Rep. 2020 Sep 9;10:14859. doi: 10.1038/s41598-020-71844-8 (PMC7481313; doi:10.1038/s41598-020-71844-8)
Supplement: Supplementary file 1 — Supplementary Information. [file 41598_2020_71844_MOESM1_ESM.docx]

**Supplementary information**

**Pluripotent stem cell model of Shwachman–Diamond syndrome reveals apoptotic predisposition of hemoangiogenic progenitors**

Takayuki Hamabata, Katsutsugu Umeda, Kagehiro Kouzuki, Takayuki Tanaka, Tomoo Daifu, Seishiro Nodomi, Satoshi Saida, Itaru Kato, Shiro Baba, Hidefumi Hiramatsu, Mitsujiro Osawa, Akira Niwa, Megumu K. Saito, Yasuhiko Kamikubo, Souichi Adachi, Yoshiko Hashii, Akira Shimada, Hiroyoshi Watanabe, Kenji Osafune, Keisuke Okita, Tatsutoshi Nakahata, Kenichiro Watanabe, Junko Takita, and Toshio Heike

**Supplementary Table 1. List of primers**

**Supplementary Table 2. List of antibodies**

| Immunostaining | | |
| --- | --- | --- |
| Antibody Name | Company | Dilution |
| Anti-lactoferrin | Santa Cruz Biotechnology(sc-14434) |  |
| Anti-CD31 | eBioscience(14-0319-82) |  |
|  |  |  |
| Western blotting | | |
| Antibody Name | Company | Dilution |
| Anti-SBDS | Santa Cruz Biotechnology (sc-271350) |  |
| Anti-TF2B | Santa Cruz Biotechnology (sc-225) |  |
|  |  |  |
| FACS | | |
| Antibody Name | Company | Dilution |
| CD34 (PE) | Beckman Cooulter (A07776) | 1:50 |
| CD34 (Brilliant Violet) | BD Bioscience (562577) | 0.111111 |
| CD45 (APC) | BD Bioscience (340943) |  |
| CD45 (FITC) | eBioscience (11-9459-42) |  |
| KDR | Relia Tech GmbH (101-MBi20) |  |
| CD31 | Biolegend (303104) |  |
| VE-Cadherin | BD Bioscience (580410) |  |
| CD133 | Miltenyi Biotec (130-090-826) |  |
| CD141 | Biolegend (344105) |  |
| CD146 | Miltenyi Biotec (130-092-853) |  |
| BrdU | BD Bioscience (555627) |  |
| p53 (pS37) | BD Bioscience (560282) |  |

**Supplementary Figure S1.** (a) Human ESC-like morphology of SDS-iPSCs. Scale bar: 400 μm. (b, c) DNA sequencing analysis of *SBDS* RT-PCR product (b) and karyotype analysis (c) of SDS and control iPSCs. (d) Expression of *OCT3/4*, *Lin28*, and *L-Myc* in SDS and control iPSCs. One primer set detects only the transgene (black bars), whereas the other detects both the transgene and endogenous gene (white bars). (e) Teratoma formation from SDS and control iPSCs in NOD/SCID/γc^null^ mice. Arrows indicate endoderm (respiratory epithelium), mesoderm (cartilage), and ectoderm (pigmented epithelium). Scale bar: 200 μm.

**Supplementary Figure S2.** (a) May–Giemsa staining of floating HCs obtained from SDS-iPSC clones (SDS2 and SDS3) transduced with SBDS or empty vector on day 30 of differentiation. obtained from SDS and control iPSCs on day 30 of differentiation. Scale bar: 100 μm. (b, c) Myeloperoxidase (MPO) staining (b) and immunostaining for lactoferrin (LCT, c) of floating HCs derived from SDS and control iPSCs. (d) Bactericidal activity of floating HCs derived from SDS-iPSC and control iPSCs. (e) Sequential analysis of the number of floating HCs generated from SDS-iPSC clones (SDS1-2, SDS2, and SDS3) transduced with SBDS or empty vector. (f, g) Morphological classification of floating HCs derived from SDS and control iPSCs (f) and from SDS-iPSC clones transduced with SBDS or empty vector (g). Cells were classified into three groups: myeloblast and promyelocyte (MB/ProM), myelocyte and metamyelocyte (Myelo/Meta), and band and segmented neutrophils (Band/Seg). Data represent means ± SEM of triplicate wells; representative results from one of three independent experiments are shown.

**Supplementary Figure S3.** (a) Sequential analysis of the number of floating HCs generated from SDS-iPSC clones transduced with SBDS or empty vector. (b, c) Representative DiI-Ac-LDL uptake assay (b) and immunophenotype (c) of CD31^+^ EC cells generated from SDS and control iPSCs. Human umbilical vein endothelial cells (HUVEC) were used as a positive control in b. (d) Tube formation by CD31^+^ EC cells generated from SDS-iPSC clones transduced with SBDS or empty vector. Data represent means ± SEM of triplicate wells; representative results from one of three independent experiments are shown.

**Supplementary Figure S4.** (a–c) Proportion of apoptotic cells in undifferentiated SDS-iPSCs clones transduced with SBDS or empty vector (A), and their derived neutrophils (b) and EC cells (c). (d, e) Proportion of BrdU-labeled cells among undifferentiated SDS and control iPSCs (d) and their derived hemoangiogenic progenitors (e).
